# Longitudinal patterns of health-related quality of life and dialysis modality: a national cohort study

**DOI:** 10.1186/s12882-018-1198-5

**Published:** 2019-01-08

**Authors:** Nwamaka D. Eneanya, Dugan W. Maddux, Marta M. Reviriego-Mendoza, John W. Larkin, Len A. Usvyat, Frank M. van der Sande, Jeroen P. Kooman, Franklin W. Maddux

**Affiliations:** 1 Renal-Electrolyte and Hypertension Division, Perelman School of Medicine, University of Pennsylvania, 307 Blockley Hall, 423 Guardian Drive, Philadelphia, PA 19104 USA; 2 Fresenius Medical Care North America, Waltham, MA USA; 3 Division of Nephrology, Department of Internal Medicine, Maastricht University Medical Center, Maastricht, Netherlands

**Keywords:** Dialysis, dialysis modality, quality-of-life

## Abstract

**Background:**

Health-related quality of life (HrQoL) varies among dialysis patients. However, little is known about the association of dialysis modality with HrQoL over time. We describe longitudinal patterns of HrQoL among chronic dialysis patients by treatment modality.

**Methods:**

National retrospective cohort study of adult patients who initiated in-center dialysis or a home modality (peritoneal or home hemodialysis) between 1/2013 and 6/2015. Patients remained on the same modality for the first 120 days of the first two years. HrQoL was assessed by the Kidney Disease and Quality of Life-36 (KDQOL) survey in the first 120 days of the first two years after dialysis initiation. Home modality patients were matched to in-center patients in a 1:5 fashion.

**Results:**

In-center (*n*=4234) and home modality (*n*=880) patients had similar demographic and clinical characteristics. In-center dialysis patients had lower mean KDQOL scores across several domains compared to home modality patients. For patients who remained on the same modality, there was no change in HrQoL. However, there were trends towards clinically meaningful changes in several aspects of HrQoL for patients who switched modalities. Specifically, physical functioning decreased for patients who switched from home to in-center dialysis (*p*< 0.05).

**Conclusions:**

Among a national cohort of chronic dialysis patients, there was a trend towards different patterns of HrQoL life that were only observed among patients who changed modality. Patients who switched from home to in-center modalities had significant lower physical functioning over time. Providers and patients should be mindful of HrQoL changes that may occur with dialysis modality change.

**Electronic supplementary material:**

The online version of this article (10.1186/s12882-018-1198-5) contains supplementary material, which is available to authorized users.

## Introduction

Approximately 680,000 patients in the United States are afflicted with end-stage renal disease (ESRD) and roughly 70% of these patients receive maintenance therapies in the form of hemodialysis (HD) or peritoneal dialysis (PD).[1] Compared to the general population, dialysis patients have lower health-related quality of life (HrQoL), which is strongly associated with poorer dialysis adherence, increased hospitalizations, and higher mortality.[2–5] Importantly, although in-center HD patients and home modality patients appear to have different patterns of HrQoL, it is unclear if one modality type results in improved health status.[5–10] For instance, one study explored quality of life among 16,755 in-center HD patients and 1,260 PD patients and found no significant difference in the physical aspects of the SF-36 survey between the two groups, although PD patients scored higher on mental aspects.[8] Notably, this study focused on cross-sectional relationships between HrQoL and dialysis modality.

Several studies have compared HrQoL changes over time between incident ESRD patients receiving different renal replacement therapy modalities (e.g., in-center HD, home dialysis, and renal transplantation), however most have featured small sample sizes, shorter follow-up times, or have primarily focused on stable modality choices over time.[11–18] To our knowledge, there have not been any studies that have examined changes in HrQoL over time based on modality change among a large national cohort of chronic dialysis patients. Specifically, we investigated whether changes in HrQoL over time would be different for patients who remained on the same modality versus patients who changed modality.

## Materials and Methods

### Study population and data source

Approximately 43% of the current United States outpatient dialysis population is represented in the Fresenius Medical Care North America (FMCNA) database.[19] We utilized data from patients ≥18 years of age who received any dialysis treatment within the FMCNA network of outpatient clinics between January 1, 2013 and June 30, 2015. We included patients who had their first outpatient dialysis treatment with FMCNA within 120 days of dialysis initiation and who also completed two Kidney Disease and Quality of Life (KDQOL-36) surveys within 485 days. Patients were only included if they remained on the same treatment modality for the first 120 days of the first year and second year. Patients were categorized as using a home dialysis modality (e.g., PD [n=825] and home hemodialysis (HHD) [n=61]) or receiving in-center HD treatments (n=19,129) based on their first modality recorded in the FMCNA database. We also relied on the FMCNA database to ascertain changes in dialysis modality 1) within the first 120 days of dialysis initiation (Period 1); and 2) between 365 and 485 days after dialysis initiation (Period 2). Next, we performed chart reviews of 40 randomly selected patients to assess the accuracy of recorded dialysis modality data. Baseline data on patient demographics, comorbidities, catheter use (central venous and peritoneal), residual renal function, blood pressure, and laboratory variables were ascertained within the first 120 days of dialysis initiation using the FMCNA database. We confirmed presence of residual renal function and catheter use if these were documented on the first day of dialysis initiation. For patients who had multiple lab values within the first 120 days, mean levels were calculated and used for analysis.

### Outcomes

We used the KDQOL-36 survey to assess HrQoL for all patients who had completed at least two surveys within the study period.[20] This instrument has been used extensively to assess HrQoL among incident and prevalent dialysis patient populations and includes the Physical Component Summary (PCS), Mental Component Summary (MCS), Symptom/Problems (SPS), Burden of Kidney Disease (BKD), and Effects of Kidney Disease (EKD) subscales. For in-center patients, roughly half of all patients completed their surveys during their treatment or at home without social work assistance. The remaining patients completed their surveys during their treatments with social worker assistance. For home modality patients, approximately half of all patients brought their completed surveys to their clinics during monthly follow-up visits whereas the remaining patients completed their surveys in-person or over the phone with their clinic social worker. To ascertain changes in HrQoL over time, we reviewed KDQOL data during Period 1 and Period 2. All KDQOL surveys are maintained in FMCNA dialysis facilities and are accurate for all patients based on mandatory rules.

### Statistical analysis

Patient characteristics were described using percentages for categorical variables and mean ± standard deviation (SD) for continuous variables. To control for any confounding effects, we selected patients using a home modality and matched them individually to clinically similar in-center HD patients using nearest neighbor matching on the logit of the propensity score for sex, age, race, albumin, number of comorbidities, and presence of residual renal function. We assessed change in each KDQOL subscale score between Period 1 and 2 and reported the change as a percentage. Two-sided p values less than 0.05 were used to indicate statistical significance. All analyses were conducted using SAS software (version 9.3; SAS Institute Inc., Cary, NC) and matching was performed using the MatchIt package in R.[21]

A protocol detailing this retrospective analysis was reviewed by Schulman Institutional Review Board (IRB) in Cincinnati, OH and determined to be exempt from regulatory approval. This study was of minimal risk and did not require informed consent.[22, 23]

## Results

### Patient characteristics

**Fig. 1:**
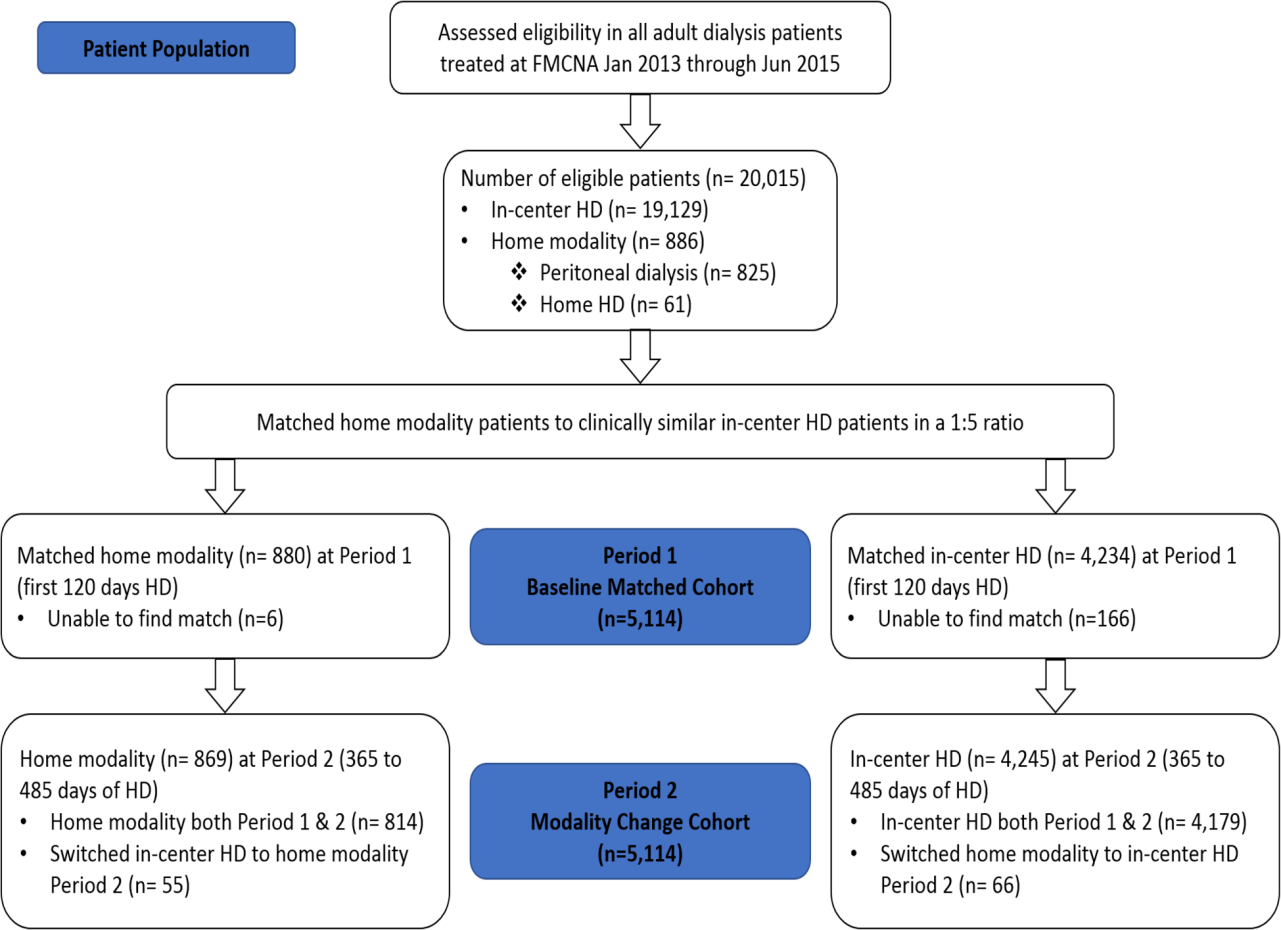
Study flow diagram

During the study period, we identified a total of 880 patients who initiated treatment with a home modality at a chronic dialysis facility affiliated with FMCNA and who met eligibility criteria. Home modality patients were matched in a 1:5 fashion to 4234 in-center patients (Figure 1). One hundred and sixty-six PD patients did not have a similar in-center patient to match with. Matching procedures resulted in relatively similar distributions of demographic and clinical variables between the two groups (Table 1). The mean age ± SD for in-center patients and home modality patients were 60.9 ±14 and 57.3 ±14.5 years, respectively (*p* < 0.01). Compared to home modality patients, there was a higher proportion of in-center dialysis patients who were of Hispanic ethnicity (11% vs. 8%, *p* < 0.01), but no differences in terms of sex or race. In-center HD patients also had less commonly attained a bachelor’s level of education (54% vs. 56%, *p* < 0.01) compared to home modality patients, however there was no significant difference in marital status or mean annual household income between the two groups.

**Table 1:** Baseline patient characteristics^*^

Variables	All (*n*=5114)	In-center (*n*=4234)	Home (*n*=880)	*p*-value
Mean age, years	60.3 ±14	60.9 ±14	57.3 ± 15	<0.01
Male	2864 (56)	2371 (56)	475 (54)	0.25
Hispanic ethnicity	563 (11)	466 (11)	70 (8)	<0.01
Black race	1176 (23)	931 (22)	220 (25)	0.07
Mean annual household income, dollars/year	50888 ± 19073	50901 ±19098	50828 ± 18960	0.92
Education (bachelors and higher)	2762 (54)	2286 (54)	493 (56)	<0.01
Married	2148 (42)	1778 (42)	396 (45)	0.10
Mean number of comorbidities	16 ± 9	17 ± 9	15 ± 10	<0.01
Cancer	276 (5)	243 (6)	33 (4)	0.01
Diabetes	3158 (62)	2711 (64)	447 (51)	<0.01
Arrhythmias	487 (10)	421 (10)	66 (8)	0.01
Congestive heart failure	1030 (20)	903 (21)	127 (14)	<0.01
Cerebrovascular disease	290 (6)	240 (6)	50 (6)	0.99
Ischemic heart disease (without myocardial infarction)	1066 (21)	946 (22)	120 (14)	<0.01
Myocardial infarction	518 (10)	446 (11)	72 (8)	0.02
Chronic obstructive pulmonary disease	440 (9)	387 (9)	53 (6)	<0.01
Drug/alcohol dependence	127 (2)	110 (3)	17 (2)	0.20
Gastrointestinal bleeding	30 (1)	28 (1)	2 (0)	0.03
Hepatitis	225 (4)	210 (5)	15 (2)	<0.01
Pneumonia	104 (2)	80 (2)	24 (3)	0.15
Other infection	852 (17)	752 (18)	100 (11)	<0.01
Hyperparathyroidism	348 (7)	273 (6)	75 (9)	0.04
Peripheral artery disease/vascular disease	580 (11)	491 (12)	89 (10)	0.19
Catheter access use	3068 (60)	2244 (53)	836 (95)	<0.01
Presence of any residual renal function	4551 (89)	3726 (88)	783 (89)	0.71
Mean serum sodium (mmol/L)	138.8 ± 2.8	138.5 ± 2.7	139.9 ± 2.9	<0.01
Mean albumin (g/dL)	3.7 ± 0.4	3.7 ± 0.4	3.7 ± 0.4	0.33
Mean hemoglobin (g/dL)	10.7 ± 0.9	10.7 ± 0.8	11 ± 1.2	<0.01
Mean systolic blood pressure (mmHg)	142.4 ± 15.9	142.5 ± 15.6	141.3 ± 22	0.48
Mean body mass index (kg/m^2^)	30.4 ± 9.1	30.4 ± 9.2	30 ± 6.3	0.34
Mean physical composite summary score (PCS)	38.6 ± 10.4	38 ± 10.3	41.1 ± 10.5	<0.01
Mean mental composite summary score (MCS)	51.7 ± 10	51.8 ± 10	51.4 ± 9.7	0.27
Mean symptom problem score (SPS)	81 ± 14.1	80.7 ± 14.3	82.7 ± 13.4	<0.01
Mean burden of kidney disease score (BKD)	54 ± 28	53.2 ± 28	58 ± 27.6	<0.01
Mean effects of kidney disease score (EKD)	77.2 ± 19.8	76.6 ± 20.1	79.7 ± 17.7	<0.01

^*^Categorical variables are presented as numbers (percentages); continuous variables are presented as mean ± standard deviation

In-center dialysis patients had a higher mean number of comorbidities versus home modality patients (17 ± 9 vs. 15 ± 10, *p* < 0.01). Furthermore, 53% of in-center dialysis patients used catheters compared to 95% of home modality patients (*p* < 0.01). Compared to home modality patients, in-center dialysis patients also had lower mean serum sodium (138.5 ± 2.7 vs. 139.9 ± 2.9, *p* < 0.01) and hemoglobin levels (10.7 ± 0.8 vs. 11 ± 1.2, *p* < 0.01). There were no differences in residual renal function, mean albumin, mean systolic blood pressure and mean body mass index between the two groups (Table 1). When comparing home modalities, a higher proportion of PD patients were older, Black, had attained bachelor’s level education, more residual renal function, higher mean serum sodium and higher mean hemoglobin levels compared to HHD patients (Additional file 1 Table S1).

In terms of baseline HrQoL, in-center patients had lower mean KDQOL scores compared to home modality patients for almost all subscales. For the PCS subscale, in-center dialysis patients scored 38 ± 10.3 (*p* < 0.01) whereas home modality patients scored 41.1 ± 10.5 (*p* < 0.01). Similarly, scores were lower on the SPS (80.7 ± 14.3 vs. 82.7 ± 13.4, *p* < 0.01) and EKD subscales (76.6 ± 20.1 vs. 79.7 ± 17.7, *p* < 0.01) for in-center versus home modality patients. The largest difference in mean scores between the two groups occurred on the BKD subscale (53.2 ± 28 for in-center vs. 58 ± 27.6 for home modality patients, *p* < 0.01). There was no difference in MCS subscale scores between the two groups (Table 1). PD patients had higher mean PCS, SPS, BKD, and EKD scores compared to HHD patients (see Additional file 1).

### Changes in HrQOL over time

Out of 40 charts reviewed, 100% of patients who stayed on the same dialysis modality accurately matched the modality listed in the FMCNA database. Additionally, 100% of patients who switched modalities matched the modalities listed in the FMCNA database. Out of 5114 patients, 4179 remained on in-center dialysis and 814 remained on home modalities. For patients who changed dialysis modalities, 55 switched from in-center to a home modality and 66 switched from a home modality to in-center dialysis.

Baseline characteristics for patients by treatment modality over time are listed in Table 2. Patients who changed from home modalities to in-center dialysis were more often of Black race, had lower annual household income, and were unmarried as compared to in-center patients who changed to home or those who remained on the same modality. Additionally, patients who switched from home modalities to in-center dialysis tended to have a higher number of comorbidities, lower albumin, higher systolic blood pressure, and higher body mass index as compared to in-center patients who changed to home or those who remained on the same modality (Table 2).

**Table 2:** Patient characteristics by treatment modality over time^*^

Variables	In-center to in-center (*n*=4179)	Home to home (*n*=814)	In-center to home (*n*=55)	Home to in-center (*n*=66)
Mean age, years	61.0 ±13.9	57.2 ±14.4	53.0 ±14.5	57.6 ±15.4
Male	2340 (56)	440 (54)	25 (45)	38 (58)
Hispanic ethnicity	460 (11)	65 (8)	3 (5)	5 (8)
Black race	919 (22)	195 (24)	11 (20)	23 (35)
Mean annual household income, dollars/year	50888 ± 19080	51101 ±19064	51864 ± 20602	47460 ±17413
Education (bachelors and higher)	2257 (54)	456 (56)	30 (55)	36 (54)
Married	1755 (42)	374 (46)	23 (42)	21 (32)
Mean number of comorbidities	17 ± 9	14 ±10	18 ±10	20 ±12
Cancer	251 (6)	33 (4)	4 (7)	1 (2)
Diabetes	2675 (64)	415 (51)	30 (55)	33 (50)
Arrhythmias	418 (10)	57 (7)	8 (15)	6 (9)
Congestive heart failure	878 (21)	122 (15)	12 (22)	7 (11)
Cerebrovascular disease	251 (6)	49 (6)	4 (7)	5 (8)
Ischemic heart disease (without myocardial infarction)	919 (22)	106 (13)	9 (16)	15 (23)
Myocardial infarction	460 (11)	65 (8)	5 (9)	5 (8)
Chronic obstructive pulmonary disease	376 (9)	49 (6)	4 (7)	5 (8)
Drug/alcohol dependence	125 (3)	16 (2)	0	2 (3)
Gastrointestinal bleeding	42 (1)	0	1 (2)	1 (2)
Hepatitis	209 (5)	8 (1)	4 (7)	3 (5)
Pneumonia	84 (2)	25 (3)	2 (4)	3 (5)
Other infection	752 (18)	90 (11)	14 (25)	11 (17)
Hyperparathyroidism	251 (6)	73 (9)	2 (4)	5 (8)
Peripheral artery disease/vascular disease	460 (11)	81 (10)	12 (22)	9 (14)
Catheter access use	2215 (53)	773 (95)	38 (69)	63 (95)
Presence of any residual renal function	3678 (88)	724 (89)	48 (87)	58 (88)
Mean serum sodium (mmol/L)	138.5 ± 2.7	139.9 ± 2.9	139.1 ± 2.7	139.4 ± 3.0
Mean albumin (g/dL)	3.7 ± 0.4	3.7 ± 0.4	3.7 ± 0.5	3.5 ± 0.4
Mean hemoglobin (g/dL)	10.7 ±0.8	11.1 ±1.2	10.7 ± 0.7	10.8 ±1.4
Mean systolic blood pressure (mmHg)	142.4 ± 15.5	140.6 ± 21.8	144.5 ± 16.6	154.4 ± 22.3
Mean body mass index (kg/m^2^)	30.4 ± 9.3	29.8 ± 6.2	30.7 ± 7.3	32.8 ± 6.9
Mean physical composite summary score (PCS)	38.0 ± 10.3	41.1 ± 10.5	38.5 ± 9.9	41.7 ± 10.4
Mean mental composite summary score (MCS)	1.8 ± 10.0	51.5 ± 9.7	51.1 ± 9.9	50.4 ± 10.2
Mean symptom problem score (SPS)	80.7 ± 14.2	82.8 ± 13.2	78.4 ± 15.0	81.3 ± 15.4
Mean burden of kidney disease score (BKD)	53.3 ± 28.0	58.0 ± 27.7	49.2 ± 30.9	57.1 ± 26.7
Mean effects of kidney disease score (EKD)	76.7 ± 20.1	79.9 ± 17.6	71.4 ± 22.8	76.9 ±19.2

^*^Categorical variables are presented as numbers (percentages); continuous variables are presented as mean ± standard deviation

Changes in mean KDQOL scores between Period 1 and Period 2 based on dialysis modality are displayed in Table 3. Overall, KDQOL mean subscale scores overtime ranged from 38.6 ± 10.4 to 81.3 ± 14.1 in Period 1 to 38.7 ± 10.7 to 80.7 ± 14.3 in Period 2. Using established criteria for minimal clinically meaningful change (3 to 5 units), [24, 25] HrQoL did not change over time for patients who remained on in-center dialysis or home modalities. However, patients who switched from in-center dialysis to home modalities had a large increase in the BKD (49.2 ± 30.9 in Period 1 to 56.1 ± 29.1 in Period 2) and EKD mean subscale scores (71.4 ± 22.8 in Period 1 to 76.9 ± 19.6 in Period 2). In comparison, patients who switched from a home modality to in-center dialysis had decreases in the PCS (41.7 ± 10.4 in Period 1 to 38 ± 10.6 in Period 2, *p* < 0.05) and BKD (57.1 ± 26.7 in Period 1 to 53.5 ± 27.6 in Period 2) mean subscale scores over time. Although there were trends towards clinically significant changes, apart from a decrease in PCS scores for patients who switched from a home modality to in-center dialysis, changes in KDQOL subscale scores over time were not statistically significant (Table 3).

**Table 3:** Changes in HrQoL over time^*^

	All (*n* = 5114)			In-center to in-cente (*n*=4179)			Home to home(*n*=814)			In-center to home(*n*=55)			Home to in-center(*n*=66)		
KDQOL subscales	Period 1	Period 2	95% CI	Period 1	Period 2	95% CI	Period 1	Period 2	95% CI	Period 1	Period 2	95% CI	Period 1	Period 2	95% CI
PCS	38.6 (10.4)	38.7 (10.7)	(-0.6, 0.3)	38 (10.3)	38.4 (10.7)	(-0.9, 0.0)	41.1 (10.5)	40.2 (10.6)	(-0.1, 1.9)	38.5 (9.9)	38.5 (10.1)	(-3.8, 3.7)	41.7^**^ (10.4)	38^**^ (10.6)	(0.1, 7.3)
MCS	51.7 (10)	52 (9.9)	(-0.6, 0.1)	51.8 (10)	52 (9.9)	(-0.6, 0.2)	51.5 (9.7)	52.1 (9.8)	(-1.6, 0.3)	51.1 (9.9)	49.1 (9.8)	(-1.7, 5.7)	50.4 (10.2)	50.8 (9.9)	(-3.9, 3.1)
SPS	81.3 (14.1)	80.7 (14.3)	(-0.2, 0.9)	80.7 (14.2)	80.4 (14.4)	(-0.4, 0.9)	82.8 (13.2)	81.6 (13.8)	(-0.2, 2.5)	78.4 (15)	80.2 (12.7)	(-7.0, 3.5)	81.3 (15.4)	82.6 (13.3)	(-6.2, 3.7)
BKD	54 (28)	54.6 (28.5)	(-1.6, 0.6)	53.3 (28)	53.8 (28.6)	(-1.7, 0.7)	58 (27.7)	58.4 (27.8)	(-3.1, 2.3)	49.2 (30.9)	56.1 (29.1)	(-18.3, 4.4)	57.1 (26.7)	53.5 (27.6)	(-5.8, 13.0)
EKD	77.2 (19.8)	77.6 (19.8)	(-1.1, 0.4)	76.7 (20.1)	77.2 (20.1)	(-1.3, 0.4)	79.9 (17.6)	79.8 (17.8)	(-1.6, 1.8)	71.4 (22.8)	76.9 (19.6)	(-13.6, 2.4)	76.9 (19.2)	77.9 (19)	(-7.6, 5.6)

^*^Continuous variables are presented as mean (± standard deviation); Period 1 = Day 0 to 120 days after dialysis initiation, Period 2 = Day 365 to 485 after dialysis initiation; *CI* Confidence interval; *KDQOL* Kidney Disease and Quality of Life, *PCS* Physical Component Summary, *MCS* Mental Component Summary, *SPS* Symptom/Problems, *BKD* Burden of Kidney Disease, *EKD* Effects of Kidney Disease. ^**^Change in score statistically significant (*p* < 0.05)

## Discussion

Among a national cohort of adult patients who initiated chronic in-center or home dialysis, HrQoL varied by dialysis modality. In-center dialysis patients had lower mean KDQOL subscale scores compared to home modality patients at baseline. For patients who remained on the same modality, there was no significant change in HrQoL over time. However, patients who switched modalities had trends towards clinically meaningful changes in certain KDQOL subscale scores. Specifically, home modality patients who switched to in-center dialysis had significantly lower physical functioning over time.

Monitoring and promoting the well-being of dialysis patients along the spectrum of their kidney disease is crucial to patient-centered care. Patients with advanced chronic kidney disease are often faced with complex decision-making (including dialysis access planning and modality choice) in the setting of poor health and functional status which could impact quality of life at dialysis initiation.[26, 27] Specifically, we demonstrated an appreciable difference in baseline quality of life between in-center and home modality incident patients, which remained stable over time if there was no change in modality. Our findings differ from previous longitudinal studies which have shown changes in HrQoL over time for patients who remain on the same dialysis modality.[12, 15] However, one recent study prospectively investigated health and functioning status (via self-reported health status and the presence of bothersome symptoms via the KDQOL-SF) among older patients receiving chronic dialysis.[17] Patients who received HHD or PD were each independently found to have decreased risk for low health status compared to those who dialyzed within clinics after 12 months of treatment. Most patients in the study were noted to have stable or improved health status over time. After dialysis initiation, patients may improve clinically and adapt to lifestyle changes which may result in similar quality of life. Furthermore, compared to in-center dialysis patients, patients on home dialysis modalities may have higher HrQoL because they feel less disruptions from their disease and are less likely to perceive themselves in a patient role.[28] Regardless of dialysis modality, closely assessing physical and mental function and effectively managing symptom burden is key to preserving patients’ HrQoL over time.[29]

Although few studies have investigated the association of patient well-being with changes in ESRD treatment [30, 31], we noted that certain aspects of quality of life changed over time depending on dialysis modality. In particular, the BKD subscale score (which addresses the burden of kidney disease on life activities, relationships etc.) appeared to increase when patients switched from in-center dialysis to home modalities and decreased when they changed from home modalities to in-center dialysis. In-center dialysis patients who switched to home modalities also appeared to be bothered less by the effects of kidney disease on their daily life as evidenced by higher EKD subscale scores. A recent systematic review of qualitative studies of dialysis patients and their caregivers noted that many viewed home hemodialysis as a modality that provides independence, flexibility, and strengthened relationships.[32] These feelings may also extend to peritoneal dialysis patients although the reverse may be true specifically for elderly and frail patients who have greater physical and cognitive dysfunction.[33, 34] Additionally, we noted a significant decrease in the PCS subscale score when patients switched from home modalities to in-center dialysis. Patients who transition from home modalities to in-center dialysis may do so because of ultrafiltration failure, infection, or access-related problems which could ultimately contribute to progressive physical limitations after loss of residual renal function.[35–37]. Indeed, we noted that patients who switched from home to in-center dialysis in this study appeared to be sicker given a higher number of comorbidities and lower mean albumin compared to the other groups of patients. We also observed that patients who switched from home to in-center dialysis were more often unmarried. Home modality patients may ultimately choose to switch to in-center dialysis after suffering from isolation or emotional distress if they do not have adequate social support.[38–40]. Given these stipulations, providers should clearly delineate the potential positive and negative health status changes that could potentially occur as patients switch dialysis modalities. Engaging in shared dialysis decision-making where the specifics of each dialysis modality are reviewed can help patient reconcile their unique strengths and weaknesses with treatment objectives.[41–43]

While our study has several strengths, there are some limitations. There may have been unmeasured confounders that introduced bias into the study. Although we confirmed different patterns of HrQoL by dialysis modality, we could not infer causality due to the observational nature of the study. Therefore, it is unclear whether changes in HrQoL occurred before or after changes in modality setting and factors driving these changes were not investigated. Also, we were unable to ascertain patient preferences for dialysis modality, and therefore could not deduce whether there was a “mismatch” of modality with patient lifestyle or why a patient ultimately received a certain modality. We acknowledge that matching more home modality to in-center dialysis patients and assessing patterns of HrQoL among those who changed modalities would have been most ideal if there had been a larger study population. Additionally, we are aware that including only patients who survived throughout the study period and also only those who completed surveys limits generalizability of the study results. Lastly, although HHD and PD patients may be distinct populations, we grouped these patients based on a desire to focus on modality setting and were therefore unable to assess transfers between the two modalities and any possible subsequent effects on HrQoL.

In conclusion, different patterns of HrQoL at the time of initiation and over time vary by dialysis modality. Home modality patients appear to have higher HrQoL compared to in-center patients and less physical functioning when switching to in-center dialysis over time. More research is needed to determine the significance of patients’ preferences for dialysis modality on HrQoL over time. However, providers and patients should be mindful of possible quality of life changes that may occur when transitioning to a different dialysis modality to ultimately optimize patients’ livelihood and dialysis experiences.

## Additional file

Additional file 1: **Table S1.** Baseline characteristics for home modality patients. Table displaying differences in baseline characteristics between home hemodialysis and home peritoneal dialysis patients. (PDF 63 kb)
