# Supplementary material for: Longitudinal patterns of health-related quality of life and dialysis modality: a national cohort study
Source: BMC Nephrol. 2019 Jan 8;20:7. doi: 10.1186/s12882-018-1198-5 (PMC6325821; doi:10.1186/s12882-018-1198-5)
Supplement: Supplementary file 1 — Table S1. Baseline characteristics for home modality patients. Table displaying differences in baseline characteristics between home hemodialysis and home peritoneal dialysis patients. (PDF 63 kb) [file 12882_2018_1198_MOESM1_ESM.pdf]

Table S1. Baseline characteristics for home modality patients*

Variables	Home PD (n-=819)	Home HD (n=61)	p-value
Mean age (years)	57.3 (15)	56.1 (14)	<0.01
Male	53	69	0.09
Hispanic ethnicity	8	16	0.01
Black race	26	15	0.03
Mean annual household income (dollars/year)	50840 (19234)	50670 (14937)	0.93
Education (bachelors and higher)	56	53	<0.01
Married	44	52	0.19
Number of comorbidities	15 (10)	15 (11)	<0.01
Catheter access use	100	28	<0.01
Presence of any residual renal function	92	41	<0.01
Mean serum sodium (mmol/L)	140.0 (2.9)	138.9 (3.1)	<0.01
Mean albumin (g/dL)	3.7 (0.4)	3.9 (0.4)	0.06
Mean hemoglobin (g/dL)	11.1 (1.2)	10.5 (1.1)	<0.01
Mean systolic blood pressure (mmHg)	141.1 (22.2)	147 (16.1)	0.42
Mean body mass index (kg/m^2)	30.0 (6.2)	33.0 (8.2)	0.24
Mean physical composite summary score (PCS)	41.4 (10.4)	38.3 (11.8)	<0.01
Mean mental composite summary score (MCS)	51.3 (9.8)	52.7 (8.6)	0.19
Mean symptom problem score (SPS)	82.7 (13.5)	82.4 (12.5)	<0.01
Mean burden of kidney disease score (BKD)	58.7 (27.6)	48.1 (26.2)	<0.01
Mean effects of kidney disease score (EKD)	79.8 (17.7)	78.2 (18.3)	<0.01

*Categorical variables are presented as percentages; continuous variables are presented as mean (± standard deviation).
